# Environmental Variables Including Heavy Metals Significantly Shape the Soil Bacterial Community Structure in the Tatun Volcano Group, Northern Taiwan

**DOI:** 10.1264/jsme2.ME22005

**Published:** 2022-10-21

**Authors:** David Anderson, Ying-Ping Song, Yu-Ting Wu

**Affiliations:** 1 Department of Tropical Agriculture and International Cooperation (DTAIC), National Pingtung University of Science and Technology, Pingtung 91201, Taiwan, ROC; 2 The Experimental Forest, College of Bio-Resources and Agriculture, National Taiwan University, Nantou 55750, Taiwan, ROC; 3 Department of Forestry, National Pingtung University of Science and Technology, Pingtung 91201, Taiwan, ROC; 4 Department of Biomedical Science and Environmental Biology, Kaohsiung Medical University, Kaohsiung 80708, Taiwan, ROC

**Keywords:** co-occurrence, heatmap, next-generation sequencing, relative abundance, volcanic soil

## Abstract

Recent studies suggested the presence of magma chambers from the Tatun volcano group under northern Taiwan’s surface, the result of episodic volcanism for 0.2–2.8 million years. However, the microbial community in volcanic soil has not yet been characterized. Therefore, the present study investigated the spatial distribution of microbial communities and their relationships with environmental variables, including heavy metals. Next-generation sequencing was used to analyze the microbial community structures in three areas with different land uses: Lengshuikeng (recreational area), Zhuzihu (agricultural area), and Huangzuishan (conservation area). High contents of environmental factors, such as nitrogen (0.46–1.14%) and phosphorus (2.01–13.88 ppm), were detected. Large concentrations of heavy metals, such as copper (55.90–127.60 ppm) and zinc (36.13–147.73 ppm), were found among the three sites, whereas those of lead (83.13 ppm) and chromium (48.33 ppm) were higher in the Zhuzihu area. The most prevalent phylum across all sites was *Proteobacteria*, followed by *Actinobacteria*, *Acidobacteria*, and *Chloroflexi*, while the most abundant bacterial species was *Koribacteraceae*: NA_01, followed by *Cyanobacteria*: NA. A network ana­lysis showed that *Koribacteracea*: NA_01 positively correlated with bacterial groups, including *Flavisolibacter* sp., *Oxalobacteraceae*: NA, and *Actinomycetales*: NA_01. Based on Shannon and Simpson’s diversity indices, the diversity of bacteria was significantly less in the Huangzuishan area than in the Lengshuikeng and Zhuzihu areas. Bacterial assemblages also significantly differed (*P*<0.05) among the three sites. The present results provide clear evidence to show that environmental variables, including heavy metals, are key factors affecting the bacterial community structure in volcanic soil.

The Tatun volcano group in Yangmingshan National Park, located in northern Taiwan, plays a crucial role in protecting nature and wildlife. It ranges in elevation from 200 to 1,200 meters above sea level and contains many different subtropical and warm climate zones. Underground movement caused massive collisions millions of years ago between the Philippine oceanic plate and the Eurasian continental plate. This event resulted in intense volcanic activity and lifted the Eurasian plate. Hot magma reaching 1,000°C gushed from the erupting volcano and covered tertiary sedimentary rock to form the 20 or more volcanoes that make up the volcano cluster in Taiwan’s northern coastal region. Over thousands of years, volcanic soil, which is formed by the weathering of volcanic materials (Delmelle *et al.*, 2015), has become a particular ecosystem comprising soil used in agriculture. The original rock source naturally influenced the heavy metal content of soil because of the mineralization process as well as the post-volcanic geological environment (Doelsch *et al.*, 2006).

Microorganisms play an essential role in volcanic soil ecosystems (Guo *et al.*, 2014). One of the most critical factors influencing ecosystem functions is soil microbial diversity. The microbial community composition in developed volcanic soil provides an overview of microbial mechanisms and dynamics, both taxonomically and functionally; even alterations or reductions in bacterial communities in volcanic soil may indicate a disturbance. Any changes in the microbial composition may result in significant changes in soil function (Verhulst *et al.*, 2010).

Yangmingshan National Park contains forests, villages, small towns, and agricultural land. It has a monsoon climate, with summers beginning in early June. The hallmark of the southwest monsoon is bright mornings with thunderstorms in the afternoons. In contrast, northeast monsoons bring humid rainy weather during winter.

Understanding how land use and human activities correlate with ecological impacts can provide early evidence of how particular ecosystems change. For example, between 1996 and 2007, 704 hectares of Yangmingshan National Park forest was converted into agricultural land (Huang and Lo, 2015); however, the effects of this large-scale change remain unclear. A previous study investigated the occurrence of an acidity-driven, microbial iron cycle that results in carbon (C) and nitrogen (N) fixation in the early microbial ecosystem of Miyake-Jima volcanic deposits and surface volcanic soils (Fujimura *et al.*, 2011). Furthermore, the bacterial community of volcanic soils in the Paricutín volcano have been examined (Santillana *et al.*, 2017), and the findings obtained revealed high concentrations of metals, such as iron and arsenic, at sampling stations. Another study provided insights into the spatial distribution of microbial communities at high elevations in a volcanic zone (Solon *et al.*, 2018). A geo-microbiological approach was recently applied to volcanic soils and pioneer plants (Fagorzi *et al.*, 2019).

Although the volcanic soils examined in previous studies may have different characteristics, their microbial community structures were all shaped by particular volcanic soil variables. A previous study was performed on the archaeal community in Yangmingshan National Park using TRE-RFLP, FISH, and microscopic observations (Ng *et al.*, 2005). However, the present study is one of the first to use next-generation sequencing (NGS) to reveal the bacterial community structure and diversity in volcanic soil.

We herein investigated the spatial distribution of microbial communities in Tatun volcanic soil. Volcanic soil samples from three different land-use types were also analyzed to examine the effects of human activities on the soil bacterial structure and diversity. Three study sites—Huangzuishan (HZS), Lengshuikeng (LSK), and Zhuzihu (ZIH)—were selected. The soil at each site is classified as volcanic soil. Each site has a specific land use: HZS is associated with a conservation area, ZIH has small-scale agriculture activity, including the planting of vegetables, and‍ ‍LSK has walking/hiking trails for tourists. LSK and ZIH‍ ‍are both used for recreation. The chemical characteristics of volcanic soil, including heavy metals, were investigated to elucidate their influence on soil bacterial communities. The present study assumed that environmental factors and heavy metals significantly altered the bacterial community structure and diversity in volcanic soil in the Tatun volcano group.

## Materials and Methods

### Geographic descriptions of the study site and sampling procedures

**Fig. 1.**
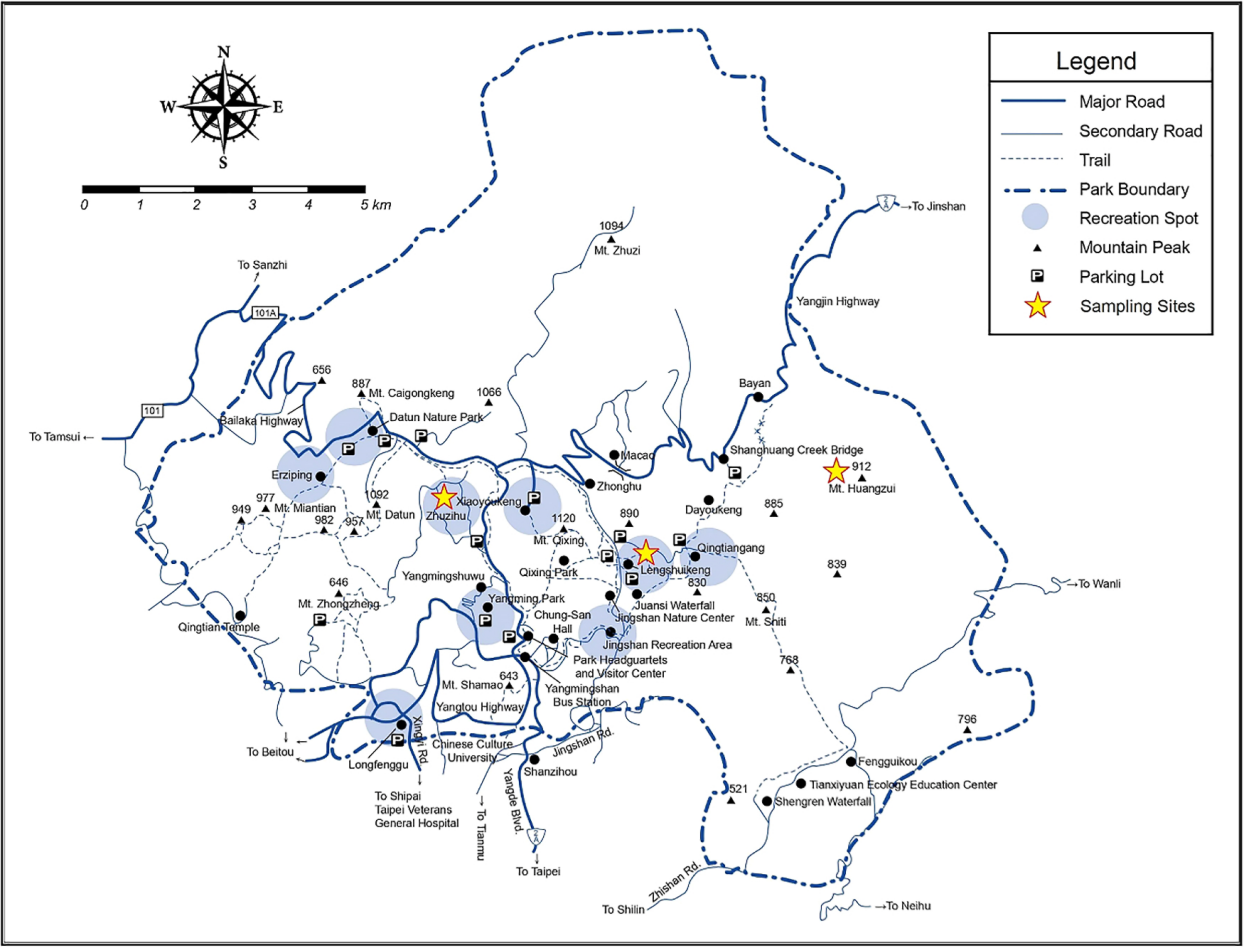
Map of Yangmingshan National Park (www.ymsnp.gov.tw).

Sampling was conducted in the Tatun volcano group (Fig. 1) and covered Lengshuikeng (LSK) (25°09′55 N, 121°33′50 E), Zhuzihu (ZIH) (25°10′31 N, 121°32′12 E), and Huangzuishan (HZS) (25°10′36 N, 121°36′22 E). We selected the center of each sampling site and established one plot of 20×20 m^2^. Each replicate was randomly selected, with 5‍ ‍m of space given between each replicate. The three areas generally have similar weather conditions. LSK and ZIH: partly cloudy, temperature: 25/32°C, wind: 16‍ ‍km h^–1^ SE, annual rainfall: 0.8‍ ‍mm, humidity: 73%, cloud cover: 28%, pressure: 1,009 mbar; HZS is similar, but with less rainfall than the other two sites. Vegetation groups in Yangmingshan National Park are divided into mountain ridge grasslands, temperate evergreen broadleaf forests, and subtropical monsoon rain forests. The national park contains 1,360 vascular bundle plants species, including dark-spotted cherry (*Mori cleyera*), Taiwan cherry (*Prunus campanulata*), formosan sweet gum (*Liquidambar formosana*), Large-leaved *Machilus* (*Machilus kusanoi*), and Red *nanmu* (*Machilus thunbergii*). LSK is a popular resting place for hiking or touring the national park. ZIH is a typical rural settlement in which clouds and fog often cover the mountain. HZS is a conservation area. Buffaloes often graze around a small pond in the valley of HZS. The total area of Yangmingshan National Park is ~11,455 hectares.

Soil samples were randomly collected from a depth of 0–10‍ ‍cm with at least three replicates. In LSK, volcanic soil samples were collected from four locations around a service station. Some of the individual soil samples for the microbial ana­lysis were collected in sterilized Falcon^®^ 50-mL tubes, placed in liquid N for several s, and then transferred and stored on dried ice until their arrival at the laboratory and storage at –20°C. The remaining soil samples were air-dried for subsequent soil chemical ana­lyses.

### DNA extraction, bacterial amplicon libraries, and DNA sequencing

The universal primer pair 968F/1391R was used to amplify the V6–V8 region in the 16S rRNA gene and generate an amplicon library. PCR reagents consisted of 25‍ ‍μL Taq Master Mix (MD Bio), 2.5‍ ‍μL (20‍ ‍mg mL^–1^) bovine serum albumin (BSA), 0.2 M each primer, and 2–5‍ ‍μg template to a total volume of 50‍ ‍μL. One hundred nanograms of diluted DNA was loaded into each well to promote amplification. The PCR program consisted of initial denaturation at 95°C for 3‍ ‍min, followed by 30 cycles at 95°C for 20‍ ‍s, 52°C for 10‍ ‍s, and 72°C for 45‍ ‍s, and then at 72°C for 5‍ ‍min, with cooling at 4°C. A 1% agarose gel with 1× TAE buffer and the PCR products was examined by SYBR^®^ Green I. The QIAEX II Gel Extraction Kit (QIAGEN) was used to purify target DNA from the gel fragment (approximately 415 bp in size). A NanoDrop spectrophotometer (Thermo Scientific) was used to check DNA quality, purity, and concentrations. Each tag was added to the 5′ ends of the 968F/1391R primer for each sample in the following PCR process. The PCR mixture contained 25‍ ‍μL Taq Master Mix (MD Bio), 2.5‍ ‍μL (20‍ ‍mg mL^–1^) BSA, 0.4 M each of the tagged primers, and 100‍ ‍ng V6–V8 amplicon to a final volume of 50‍ ‍μL. The PCR program for tag addition consisted of an initial denaturation at 95°C for 3‍ ‍min, followed by 20 cycles at 95°C for 20‍ ‍s, 52°C for 10‍ ‍s, and 72°C for 30‍ ‍s, with a final step at 72°C for 2‍ ‍min and then cooling at 10°C. The PCR product was purified and 200‍ ‍ng of the mixture from the tagged V6–V8 region was subjected to the Illumina MiSeq 2000 sequencing system (San Diego) at Genomics (Taipei).

### Analysis of soil chemical properties

Exchangeable cations are positively charged ions that are loosely attached to the edge of clay particles or organic matter (OM) in soil (Thomas, 1983). Cations include calcium (Ca^2+^), magnesium (Mg^2+^), potassium (K^+^), and sodium (Na^+^). In this case, these exchangeable cations are the total number of cations in soil that are available for direct uptake by microorganisms. Soil exchangeable cations (Ca^2+^, Mg^2+^, K^+^, and Na^+^) were assessed by atomic absorption spectrophotometry (AAS) using previously reported procedures (Chen, 2000). Volcanic soil is a rich source of environmental factors, including heavy metals such as arsenite (As), chromium (Cr), copper (Cu), nickel (Ni), lead (Pb), and zinc (Zn). Heavy metals occur in soil in a soluble form and combined state (Chiroma *et al.*, 2014). However, only soluble, exchangeable, and chelated metal species in soils are mobile and, thus, more available to plants/organisms (Hector *et al.*, 2011) (*i.e.*, extracted by 0.1 M HCl) (Wang *et al.*, 1994). The cation exchange capacity (CEC) is the total capacity of a soil to hold exchangeable cations. Based on trace elements of heavy metals in the world’s soil, heavy metal concentrations are generally higher in Yangmingshan National Park than in other countries, such as Italy and Japan, while heavy metal contents are lower than in other countries, including Germany and the USA (EPA/ROC, 1998). Available phosphorus was measured using a spectrophotometer; CEC was assessed using the Kjeldahl N device (Rhoades, 1983). Total soil N was measured using the Semimacro Kjeldahl method (MacDonald, 1977), soil OM was evaluated by wet oxidation using the Walkley-Black procedure (Nelson and Sommers, 1996), and soil heavy metals (As, Cr, Cu, Ni, Pb, and Zn) were analyzed using AAS using previously reported procedures (Baker and Suhr, 1983; Shraim *et al.*, 1999). However, we did not examine Cr(IV), Cr(VI), or As(V). Soil pH was measured using a WTW Multi 3510 IDS portable meter (Weilheim).

### Bioinformatics and statistical ana­lysis

Descriptive statistics were used to obtain a preliminary understanding of data, and a one-way ANOVA ana­lysis was then performed to test various hypotheses (Tukey’s test). High-quality and effective sequences that met the requirements were used to elucidate the bacterial community structure. The original line obtained by sequencing for chimeras and removed from ambiguous bases (N), mismatched primers, incomplete barcodes, and overpasses were filtered. Sequences were shortened using the USEARCH (v8.0.1623) system’s built-in settings to merge double-ended rows into a single line, and MOTHUR (v1.35.1) was then used to test sequence quality, keeping sequences with (1) base pair length of 400–450 bp, (2) a homopolymer not exceeding 8 bp, (3) no ambiguous bases, and (4) an average quality score greater than 20. UCHIME was used to detect and remove chimera in USEARCH software with a reference model and minimum ambiguity of 3%. Lines with 97% similarity were classified into operational taxonomic units (OTUs), showing a good representation of diversity. The UPARSE method was used to analyze OTUs (Edgar, 2013) that were subjected to quality testing and non-chimera sequences to generate a sample sequence similarity of 97%. The USEARCH global comparison was used to search for the most stable classification result of the OTU sequence in the Greengenes 13_5 gene library. Data were then organized and the relative abundance of bacteria among different samples was plotted. We measured the composition of bacterial communities among volcanic soils. Several bacteria are unknown species (NA: not available); unknown species may still be associated with a higher classification level, such as genus, family, order, class, and even phylum. Therefore, the use of NA after the name of these higher classification levels indicated the species level for the unknown bacterium. Statistical ana­lyses of the results obtained were performed using R Statistics (4.1.0) and ana­lyses of the richness index, chao1, ACE, Shannon, and Simpson’s index. The Bray-Curtis coefficient was used to obtain a dissimilarity matrix based on the preliminary ranking index ana­lysis and log (x+1) transformed abundance data. The Non-metric Multidimensional Scaling (NMDS) function of the vegan 2.5–7 package based on the Bray-Curtis distance was used to perform an NMDS ana­lysis in order to visualize the distribution of bacterial communities. The software packages igraph, ggnetwork, ggcorr, and cowplot were used to draw a network and examine relationships. The envfit function and 999 permutations were used to identify the environmental variables that had a significant impact on the bacterial community composition. The software packages pheatmap and gplots were also used to draw a heatmap and assess the relative abundance of dominant bacteria among different samples.

## Results

### Soil chemical features

Soils were rich in heavy metals, such as As, Cr, Cu, Ni, Pb, and Zn (Table 1). The concentrations of As (16.40–26.33 ppm), Pb (36.60–83.13 ppm), and Cu (55.90–127.60 ppm) were high at all three sites. The concentrations of Zn (36.13–147.73 ppm), Pb (36.60–83.13 ppm), and Cr (19.13–48.33 ppm) were the highest in ZIH. On the other hand, Cr was not detected in HZS. The concentration of Ni was high in HZS, but was not detected at any other locations. The heavy metal Ni in all samples from LSK/ZIH was not detectable at 0.00 ppm. The concentration of Ni in samples from HZS varied from not detectable (0.00 ppm) to 76 ppm, with an average concentration of 26.20 ppm. Notably, the heavy metal Ni was not detectable at 0.00 ppm in three out of five samples from HZS; therefore, Ni concentrations across all study sites did not significantly differ. Phosphate (P) concentrations (2.01–13.88 ppm) in ZIH significantly differed from those in the other areas. The concentrations of OM (6.29–9.61%) across all sites did not significantly differ, whereas N was significantly higher in HZS (1.14%) than in ZIH/LSK (0.46–0.53%). pH was the lowest (3.76) in LSK (Table 1).

### Significant differences in the bacterial community structure and diversity among sites

**Fig. 2.**
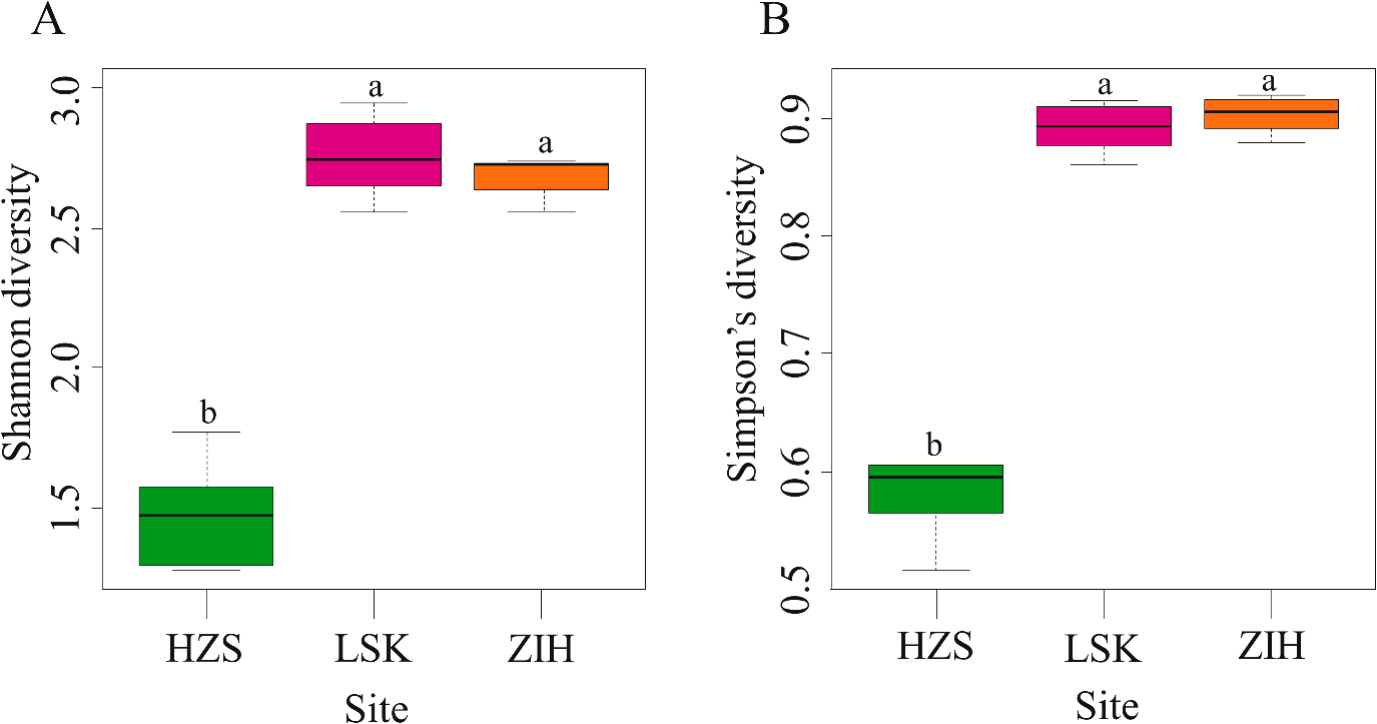
Soil bacterial diversity indices of Shannon (A) and Simpson (B). HZS, Huangzuishan area; LSK, Lengshuikeng area; ZIH, Zhuzihu area.

**Fig. 3.**
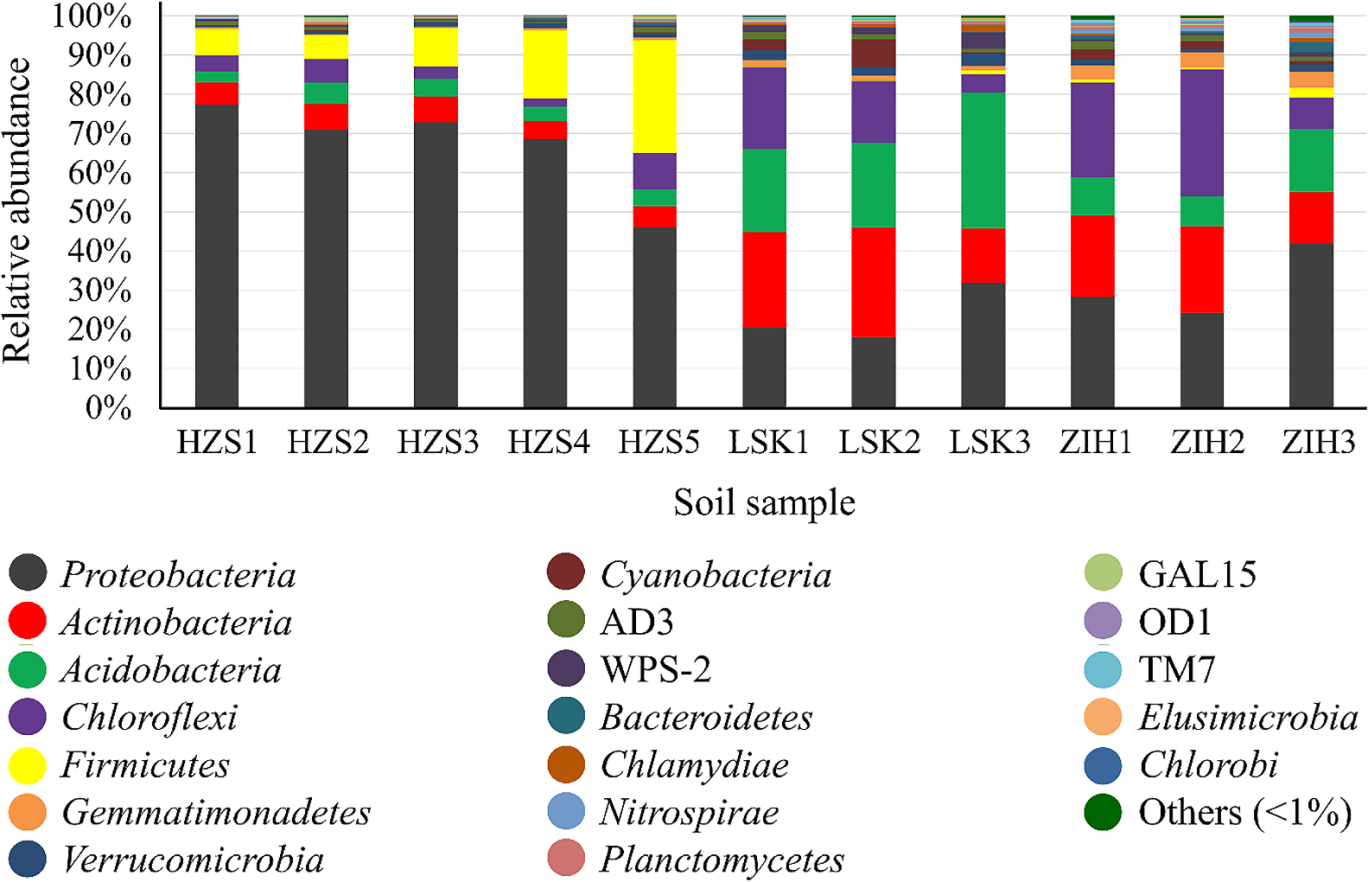
Relative abundances of dominant bacteria in soil at the phylum level (>1%). HZS, Huangzuishan area; LSK, Lengshuikeng area; ZIH, Zhuzihu area.

**Fig. 4.**
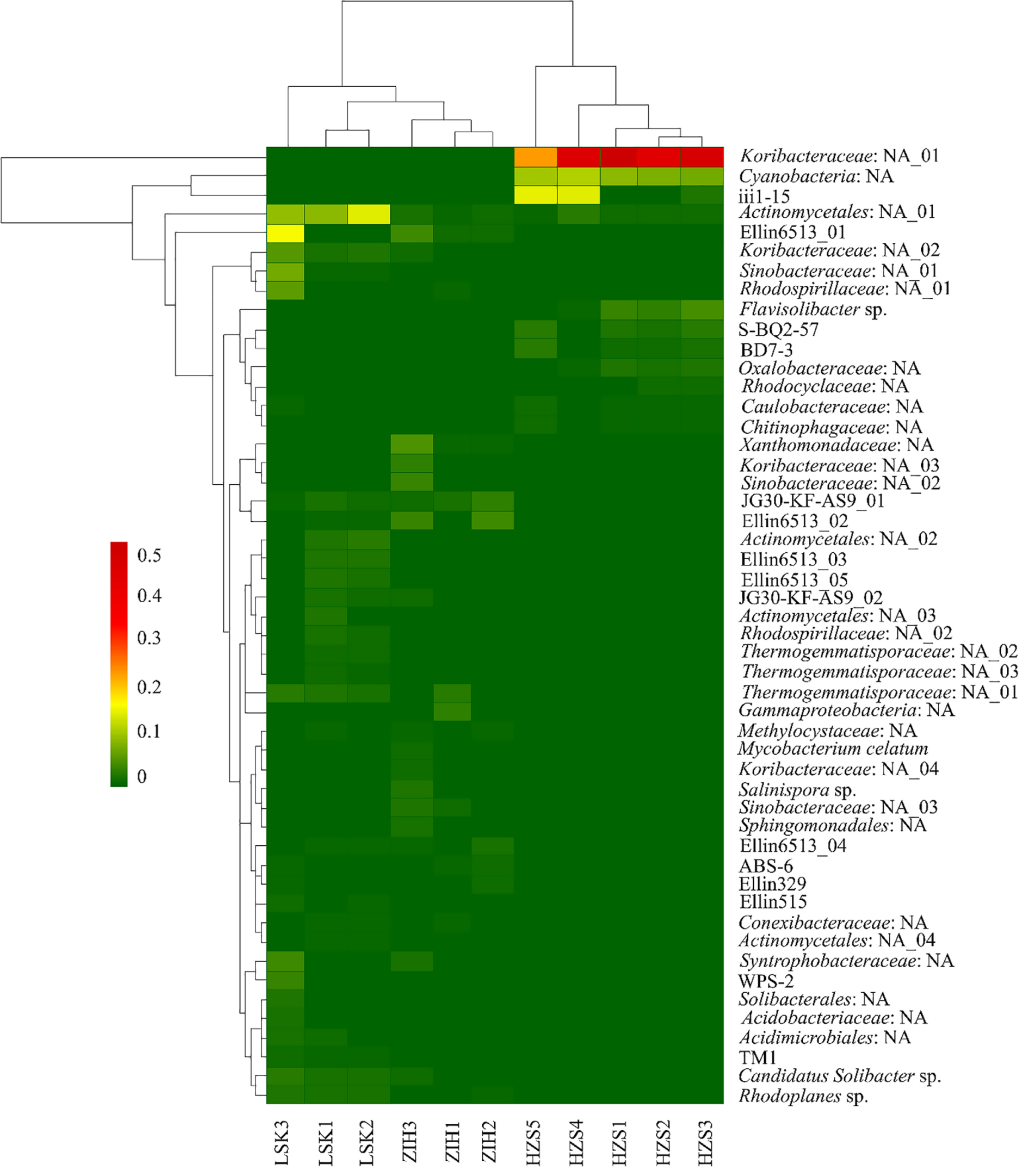
Heatmap ana­lysis of relative abundance of top 50 dominant bacteria (43.49% of *Koribacteraceae*: NA_01) at the species level across three sites. HZS, Huangzuishan area; LSK, Lengshuikeng area; ZIH, Zhuzihu area.

A total of 1,683,367 high-quality bacterial sequences were obtained and assigned to 58 bacterial phyla, 185 classes, 386 orders, 633 families, and 9,618 species. Variations among sampling sites were as follows: 20,505 to 288,135 sequences were obtained from HZS, 158,997 to 244,112 sequences from ZIH, and 62,575 to 364,462 se­quences from LSK. An ana­lysis of Shannon and Simpson’s diversity indices revealed that bacterial diversity was signifi­cantly lower in HZS than in the other areas. Soil in LSK had the highest Shannon diversity index value. The highest Simpson’s diversity index value (0.92) was found in ZIH (Fig. 2). *Proteobacteria* was the most predominant phylum across all‍ ‍three sites, followed by *Actinobacteria*, *Acidobacteria*, *Chloroflexi*, and *Firmicutes* (Fig. 3). *Proteobacteria* had a high relative abundance associated with HZS soil samples. On the other hand, *Actinobacteria* and *Acidobacteria* were associ­ated with LSK and ZIH soil, respectively. The dominant bacteria at the species level were mostly unknown species (Fig. 4), specifically *Koribacteraceae*: NA_01, *Cyanobacteria*: NA, iii1-15, *Actinomycetales*: NA_01, and Ellin6513_01 (Fig. 4). The heatmap showed that the dominant bacterial assemblages of LSK were more similar to ZIH. In HZS, *Koribacteraceae*: NA_01 was the most predominant bacterium, followed by *Cyanobacteria*: NA, and iii1-15; all three only appeared in HZS. The clustering of *Actinomycetales*: NA_01 was associ­ated with LSK and rarely appeared in the other areas, while the clustering of Ellin6513_01 was associated with ZIH and LSK (Fig. 4).

### Co-occurrence networks of bacterial groups

**Fig. 5.**
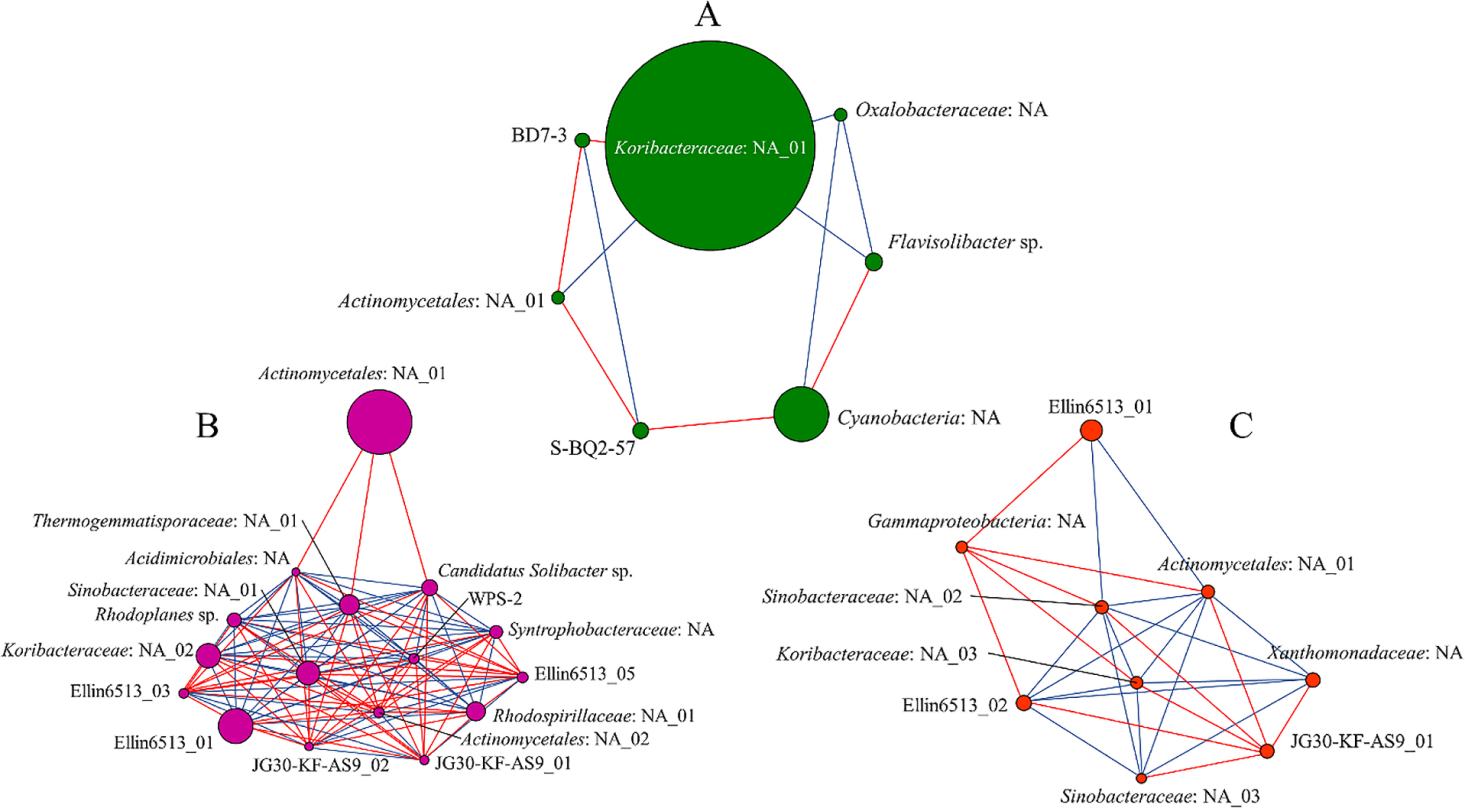
Network ana­lysis of correlations between the soil bacterial community structure at the species level in three different areas of Huangzuishan (A), Lengshuikeng (B), and Zhuzihu (C). The size of each node (vertex) represents relative abundance. The length of the edge represents the absolute value of correlations. Positive correlations are represented by blue lines and negative correlations by red lines.

The network ana­lysis revealed positive and negative correlations among bacteria in volcanic soils at the species level (Fig. 5). Blue edges indicate a positive correlation and red edges a negative correlation. The size of each vertex represents relative abundance, and the length of each edge indicates the critical value of the correlation. The networks of all three sites had different topologies, with the highest number of correlated potential bacteria being associated with LSK, followed by ZIH and HZS. *Actinomycetales*: NA_01 and Ellin6513_01 were the most prevalent bacteria in LSK and ZIH, respectively (Fig. 5). Moreover, the network in HZS had the lowest number of bacteria that positively and negatively correlated with each other; however, HZS had the highest relative abundance of *Koribacteraceae*: NA_01 (43.49%) among all sites. *Koribacteraceae*: NA_01 positively correlated with *Flavisolibacter* sp. (2.67%), *Oxalobacteraceae*: NA (1.49%), and *Actinomycetales*: NA_01 (1.44%); in contrast, *Koribacteraceae*: NA_01 negatively correlated with BD7-3 (1.52%) (Fig. 5A). The highest relative abundance in LSK was *Actinomycetales*: NA_01 (11.35%), which negatively correlated with *Thermogemmatisporaceae*: NA_01 (2.33%), *Candidatus Solibacter* sp. (2.12%), and *Acidimicrobiales*: NA (1.03%) (Fig. 5B). The highest relative abundance of ZIH was Ellin6513_01 (2.66%), which positively correlated with *Sinobacteraceae*: NA_02 (1.32%) and *Actinomycetales*: NA_01 (1.23%); in contrast, Ellin6513_01 negatively correlated with *Gammaproteobacteria*: NA (Fig. 5C).

### Environmental variables including heavy metals drive the microbial community composition

**Fig. 6.**
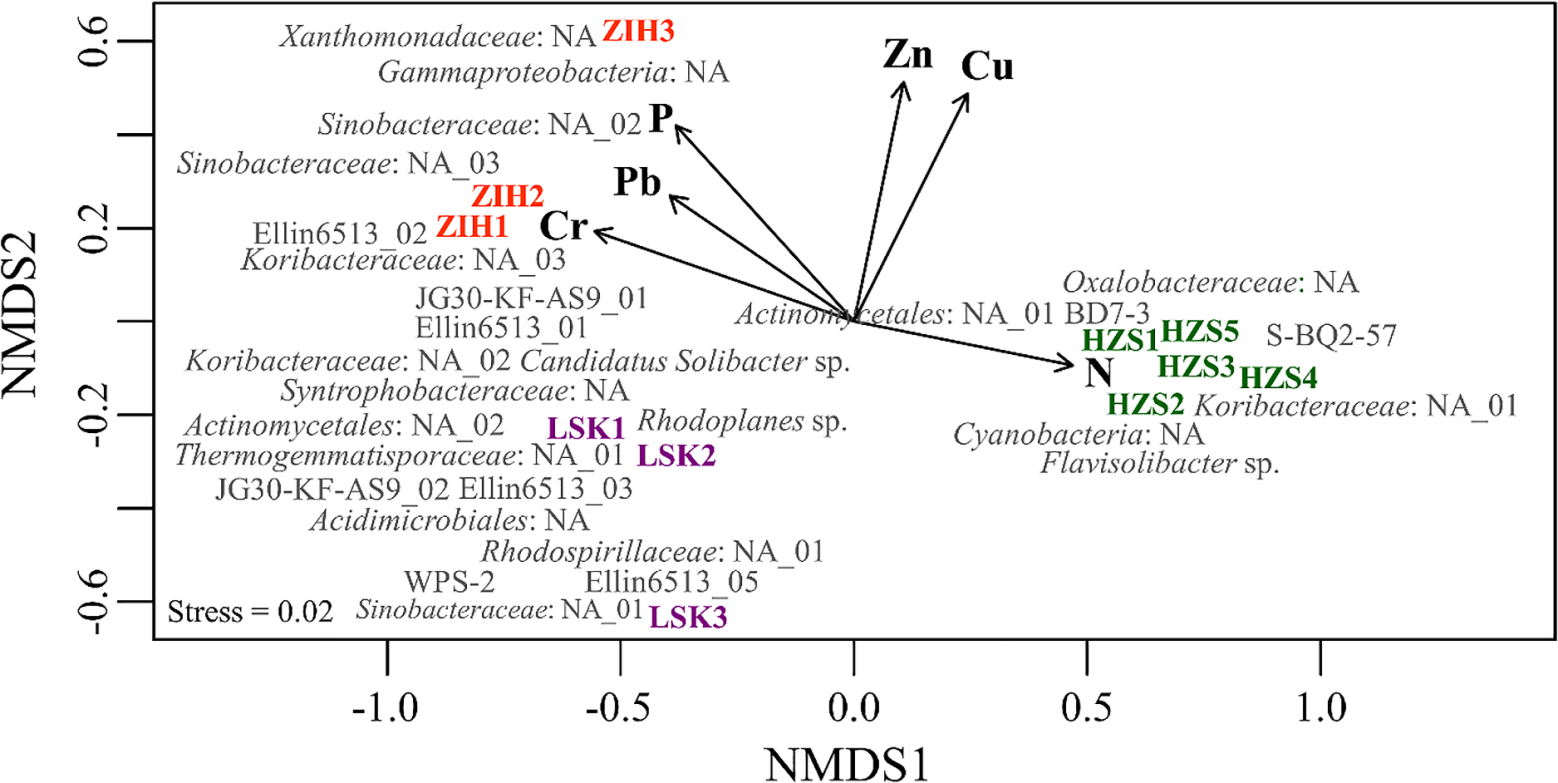
NMDS ordination of three sites based on the soil bacterial community structure at the species level in the Tatun volcano group. Environmental factors including heavy metals with significant goodness of fit based on post-hoc correlations (*P*<0.05) are presented as vectors. HZS, Huangzuishan area; LSK, Lengshuikeng area; ZIH, Zhuzihu area.

NMDS revealed a relationship between the soil bacterial community structure and environmental variables, including heavy metals, across the three sites (Fig. 6). Heavy metals, such as Cr, Cu, Pb, and Zn, significantly shaped the bacterial community structure in volcanic soils. However, heavy metals such as As and Ni did not appear in NMDS because their concentrations among the three sites did not significantly differ. Environmental factors, such as P and N, correlated with the bacterial community structure, specifically in ZIH and HZS, respectively.

## Discussion

Volcanism is a significant process of soil formation, providing a model for investigating the role of pioneer microbes. The scientific community has an interest in volcanic environments. The role of microbes in the formation of new soils, followed by the development of terrestrial ecosystems, is one of the main topics of soil biology and general ecology. The Tatun volcano group is considered to have last erupted approximately 20 ka (Chen and Lin, 2002); it had many chemical deposits in the early 20th century (Davidson, 2010). Since 2004, fumarole gas outlets have increased (Lee *et al.*, 2008). A recent study suggested the presence of magma chambers in northern Taiwan (Ohba *et al.*, 2010). Volcanic soil in the Tatun volcano group has developed with vegetation cover. Several bacterial groups were found to increase in response to changes in vegetation cover from grass to shrubs (Guo *et al.*, 2014); the vegetation structure was shown to significantly alter the microbial community composition through carbon inputs from root exudates and litter deposits (Bardgett *et al.*, 2005). Therefore, the Tatun volcanic group represents an extreme environment inhabited by lifeforms in the volcanic environment that are adapted to vegetated and developed volcanic soil. The present study examined three locations as diverse hot spots for bacteria that thrive in developed soil from the same origin of extreme environments in one national park. After thousands of years of development, these three sites may have different bacterial communities, which were examined herein. The results obtained will contribute to the discovery of new taxa, novel metabolic capacities, and the occurrence of bacterial networks; therefore, the volcanic soil of the Tatun volcano group was selected for this study. To the best of our knowledge, the volcanic soil of the Tatun volcano group in Taiwan has never been characterized, such as its bacterial diversity, using high-throughput pyrosequencing, which is crucial for bacterial identification.

Chemical properties showed different values in different regions; a previous study that did not use NGS reported a similar pattern (Santillana *et al.*, 2017). Community composition ana­lyses of the three sampling areas showed that biodiversity was lower in HZS than in LSK and ZIH (Fig. 2). The bacterial Shannon diversity index value was significantly higher in LSK than in HZS (*P*<0.05). The Shannon diversity index value was significantly higher in ZIH than in HZS (*P*<0.05). No significant differences were observed in Shannon diversity index values between ZIH and LSK (*P*>0.05). HZS had the lowest Shannon diversity index value (Fig. 2A). Simpson’s diversity index showed that the bacterial diversity profiles of ZIH and LSK did not significantly differ (Fig. 2B), which may have been due to these two areas being used as recreational spots, whereas HZS is a conservation spot. A previous study demonstrated that soil disturbed by land use had higher alpha diversity indices than soil with natural or more minor disturbances (Osburn *et al.*, 2019). Biodiversity performs essential ecological services (Altieri, 1999). However, functional diversity is essential for ecosystem productivity and stability because it increases the capacity of soil microbes to sustain soil processes (Caldwell, 2005). Although diversity and evenness were both lower in HZS volcanic soils, these reductions did not appear to impair the functions of bacterial groups. Microbial communities in ZIH and LSK may be more diverse because of interactions indicating their metabolic flexibility to heavy metals.

Analyses of LSK and ZIH sample libraries revealed the abundance of OTUs associated with *Proteobacteria*, *Actinobacteria*, *Acidobacteria*, and *Chloroflexi*. We found a higher quantity of *Proteobacteria*, followed by *Firmicutes*, *Actinobacteria*, *Chloroflexi*, and *Acidobacteria* in HZS (Fig. 3). This was consistent with the findings of previous studies that examined bacterial communities at the phylum level, such as *Proteobacteria, Actinobacteria*, and *Firmicutes* (Singh *et al.*, 2014; Fagorzi *et al.*, 2019). Several members of these phyla exist in various environments, confirming their capacity to adapt to a wide range of environments.

The heatmap displayed a clear overview of the distribution of bacterial groups in the three areas. The distribution of the retrieved sequences at the species level revealed differences in bacterial diversities among the three areas. The majority of bacteria at the species level were unknown species, such as *Koribacteraceae*: NA_01, *Cyanobacteria*: NA, and *Actinomycetales*: NA_01 (Fig. 4). These results were consistent with previous findings showing that the structure of prokaryotic communities in volcanic environments was dependent on local chemical characteristics (Tobler and Benning, 2011). *Koribacteraceae*: NA_01 was prevalent in HZS. *Actinomycetales*: NA_01 was the only bacterial group to appear in all three areas. Most groups only appeared in one area. For example, *Koribacteraceae*: NA_01, *Cyanobacteria*: NA, and iii1-15 appeared in HZS, but not in ZIH or LSK. However, JG30-KF-AS9_01 and *Thermogemmatisporaceae*: NA_01 appeared in ZIH and LSK, but were absent in HZS. Moreover, LSK and ZIH had the same bacterial groups at the species level, such as JG30-KF-AS9_01 and Ellin6513_01, which may be attributed to their land being used in similar ways.

In the present study, we compared bacterial compositions among the three areas. We performed network ana­lyses to investigate whether the bacterial community structures of each site positively and negatively correlated with each other. LSK had a more diverse primary taxonomy than the other two areas; it showed more positive and negative bacterial interactions among bacterial groups. Positive correlations among bacteria represent co-occurrence, while negative correlations indicate competition (Fig. 5). HZS displayed the lowest number of bacterial groups with positive and negative correlations (Fig. 5A). The most prevalent bacterium in LSK was *Actinomycetales*: NA_01, which also appeared in the other locations. The assumption of a positive correlation mechanism indicates mutualistic and commensal interactions between groups of bacteria. On the other hand, a negative correlation implies a parasitic interaction between groups of bacteria (Caldeira *et al.*, 2008). Negative ecological interactions (*i.e.*, competition) increase the stability of microbial communities under changing environments (Coyte *et al.*, 2015). A previous study suggested that bacteria in a disturbed system exhibited metabolic flexibility (Chen *et al.*, 2021). Similar results were obtained in the present study; the more diverse the bacterial community in LSK and ZIH, the greater their ability to adapt to disturbances (*i.e.*, land use and human activity), which was in accordance with previous findings on a grassland ecosystem (De Vries *et al.*, 2018). Based on network ana­lyses, some potential bacteria—such as JG30-KF-AS9_01 and Ellin6513_01—appeared at two sites (LSK and ZIH). *Actinomycetales* was dominant in a previous study (Riquelme *et al.*, 2015); the present results revealed that although *Actinomycetales* was not the most dominant group of bacteria, it was the only group of bacteria present in all three areas. We also showed that the highest relative abundance in the co-occurrence networks was* Koribacteraceae*: NA_01, which is consistent with a previous study that assumed that a low pH and the highest relative abundance of *Koribacteraceae* were indicators of soil disturbances (Lewis *et al.*, 2018). *Koribacteraceae* has been shown to significantly contribute to the carbon cycle (Levy-Booth *et al.*, 2019). Bacterial group iii1-15 utilizes C substrates (Liu *et al.*, 2016). Meanwhile, *Actinomycetales*: NA plays a prominent role in cycling OM (Bhatti *et al.*, 2017).

The present study provided important insights for establishing whether environmental variables, including heavy metals, affect the composition and distribution of taxa. Since the results obtained indicated a causal relationship between environmental variables, including heavy metals, and the bacterial community structure, it was possible to identify the bacterial groups affected by heavy metals and environmental factors. The findings of a study on the soil bacterial community composition in Mt. Halla, south Korea were compared those of other studies, and based on the range of different patterns observed, no unifying pattern was expected in the soil bacterial community structure or diversity trends among the world’s mountain systems (Singh *et al.*, 2014). In the present study, an ana­lysis of multivariate variations between the soil bacterial community structure and environmental variables, including heavy metals, through NMDS revealed significant separation between HZS and the other sites in NMDS1. We successfully elucidated the relationship between the most abundant bacterial compositions and environmental variables, including heavy metals. Environmental factors (N and P) including heavy metals (Cr, Cu, Pb, and Zn) significantly shaped the soil microbial community structure (Fig. 6). Based on NMDS, Cr and Pb levels generally influenced the bacterial community structure in ZIH (*P*<0.05). The abundance of heavy metals, such as Zn and Cu, positively correlated between ZIH and HZS. Heavy metals, such as As, Cr, Cu, Ni, Pb, and Zn, are potential bioaccumulative toxins from agriculture (Alloway, 1995). A study on drainage water from agricultural soils assumed that heavy metal losses occurred through erosion, with concentrations generally being low (Aldrich *et al.*, 2002); in the long term, the accumulation of heavy metals may reduce soil productivity in agricultural soils by affecting soil microbes (Birch and Davey, 1995).

NMDS showed that P concentrations were associated with ZIH, similar to previous findings (Gumiere *et al.*, 2019) with the assumption that P sources indicate soil disturbances; this was consistent with the present study, particularly in ZIH. ZIH has agriculture, but only on a small and limited scale, and soil samples were collected from the agriculture site in ZIH. ZIH showed the highest P concentration in volcanic soil. The environmental factor P is a nutrient element that influences the growth of crops because it is often deficient in soil; it is strongly absorbed by non-crystalline aluminum and iron materials, which limits its uptake by plants (Takahashi and Shoji, 2002). Although P concentrations are a limiting nutrient for most agricultural plants grown on volcanic soils, ZIH contained a large amount of plant-available P. The environmental factor P was similar to ZIH and correlated with *Xanthomonadaceae*: NA (r=0.62, *P*<0.05) and *Sinobacteraceae*: NA_03 (r=0.72, *P*<0.05). The bacterial group *Xanthomonadaceae* is an indicator for agricultural soil (An *et al.*, 2019) and has potential as an indicator regulating soil phosphorus bioavailability (Wu *et al.*, 2022). In addition, *Sinobacteraceae* was shown to be putatively involved in the release of P and uptake of C (Wong *et al.*, 2018). HZS is a conservation area consisting of a natural forest that has not been disturbed, with litterfall potentially increasing N concentrations in soil (Li *et al.*, 2011). The environmental factor N correlated with the relative abundance of the bacterium *Cyanobacteria*: NA (r=0.78, *P*<0.05). *Cyanobacteria *are photosynthetic organisms that easily survive on the minimum requirements for light, water, and carbon dioxide (CO_2_) (Castenholz, 2001). NMDS showed that environmental variables, including heavy metals, influenced the bacterial community structure and diversity of volcanic soils. All of the soil samples tested showed a clustering pattern at each site. The bacterial community structure was significantly influenced by N (*P*<0.05), and the highest value of N was associated with HZS, which is a good indicator that HZS (the conservation area) remained a natural forest. Based on the results of NMDS, we suggest that one measurement (N) may be the best predictor of the success of a conservation process in the HZS conservation area.

The correlation arrows of pH, OM, As, and Ni did not appear in NMDS, indicating that these parameters did not directly correlate with the soil bacterial community structure (Fig. 6). Large amounts of OM (humic acid, lignin, and phenolic acids) led to low mineralization rates, which reflects high total C and low total N (Hazelton and Murphy, 2007). The present study demonstrated that OM was the most abundant in LSK, but did not significantly differ from the other two locations. We found that *Gemmatimonadetes* showed low relative OTU proportions at the phylum level at the three locations. A previous study suggested that the abundance of *Gemmatimonadetes* was inversely related to soil moisture, and showed that its abundance inversely correlated with OM (DeBruyn *et al.*, 2011). In addition, Ni was not present in LSK or ZIH. Ni was only detected in HZS because plants in the natural forest require Ni in small quantities for normal development due to its role in N metabolism (De Macedo *et al.*, 2016).

In the present study, the average concentrations of Cr, Ni, Pb, and Zn were lower than the recommended maximum permissible levels of heavy metals in Taiwan (EPA/ROC, 1998). Environmental regulations for As and Cu are 10 and 35 ppm, respectively; we found that heavy metals, such as As and Cu, exceeded environmental regulations (Chen, 2000). The high concentration of As may reflect the effects of N cycling (Li *et al.*, 2022). Furthermore, the elevated level of Cu in soil may harm the ability of bacterial groups to fix N (Tindwa *et al.*, 2014), such as *Actinomycetales*: NA_01 (r=–0.85; *P*<0.05). Another study identified *Actinomycetales* as a heterotrophic denitrifying bacteria (Wang *et al.*, 2020).

Heavy metals, such as As, Cr, and Pb, are hazardous to ecosystems and humans; their concentrations fluctuated from low in HZS to high in LSK and ZIH. Average heavy metal contents were not affected by cultivation, and characteristic behaviors were maintained, except for a slight increase in Pb and Zn contents (Ahn and Chon, 2010). Excessive heavy metals, such as Zn, may be harmful to plants—*e.g.*, plants become yellow or reddish-brown with decreased yields and slow growth. The human body may be directly or indirectly harmed by soil contamination, which may have neurological consequences and cause disorders, such as cancer and skin diseases.

As a country that has been advocating environmental protection in recent years, Taiwan has properly managed the Tatun volcano group in Yangmingshan National Park and prevented pollution from various industries from entering the area. However, there are small-scale agricultural activities in ZIH. Agricultural soil in ZIH is the original soil of that location (*i.e.*, no soil was imported); after thousands of years of development, the native soil became suitable for agriculture. HZS is a conservation area. In LSK, soil samples were collected on a grassland away from a hiking trail. Soil in the three locations has the same original rock source and becomes native soil over thousands of years of development; however, land uses differ. The three different sampling sites with different usages were selected for the present study to avoid human disturbance factors on the microbial community. Therefore, the shift in the microbial community originated from natural factors. However, other studies (Ng *et al.*, 2005; Huang and Lo, 2015) conducted at different locations from our sampling sites in Yangmingshan National Park did not avoid the effects of anthropogenic activities on soil and water. Moreover, intense human activities, such as farming and building construction, continue to exist. Therefore, the present study showed changes in the bacterial community, which indicated not only the impact of natural factors, but also the indirect influence of including anthropogenic factors in the national park. We considered the sample from HZS to be the original natural community without human disturbances from the viewpoint of ecological conservation; therefore, the high possibility of changes in the microbial community in HZS originated from natural factors. LSK and ZIH showed alterations in bacterial groups from those in HZS as the baseline of our study; therefore, changes in the microbial community in LSK and ZIH with different land uses, such as urbanization and farming, may be a predictor of disturbances caused by potential anthropogenic factors. In addition, human activities deserve more attention when assessing soil because of their inevitable impact, mainly deforestation. As such, the present results provide comprehensive information for land-use and manage­ment strategies in the Tatun volcano group in Yangmingshan National Park, and the government needs to modify land development policies and plans for land-use changes in order to avoid illegal development activities.

In conclusion, volcanic soils in the Tatun volcano group in Yangmingshan National Park may have different characteristics from other volcanoes, such as the Oyama volcano in Japan and the Socompa volcano along the Chile-Argentina border; other volcanoes generally have no vegetation and are still in the form of a substrate tempered by volcanic eruptions, and are characterized by lava, tephra, and volcanic ash, fumarolic and dry soils (Fujimura *et al.*, 2011; Singh *et al.*, 2014). Volcanic soils in Yangmingshan National Park are native soil (Andisols): suitable for agriculture and contain vegetation and forests. This is the first study to examine the bacterial communities colonizing and inhabiting volcanic soil in Yangmingshan National Park, and we encourage further research on soil in this volcanic environment. We evaluated the relationships between microbial ecology and environmental variables, including heavy metals. We also described bacterial community structures in the park’s soils and found that the most abundant bacterial species was *Koribacteraceae*: NA_01 associated with HZS. Based on NMDS, we showed that environmental variables, including heavy metals, governed the bacterial community’s composition in volcanic soil. Environmental parameters, such as N, may be the best indicator of the conservation process in HZS.

## Citation

Anderson, D., Song, Y.-P., and Wu, Y.-T. (2022) Environmental Variables Including Heavy Metals Significantly Shape the Soil Bacterial Community Structure in the Tatun Volcano Group, Northern Taiwan. *Microbes Environ ***37**: ME22005.

https://doi.org/10.1264/jsme2.ME22005

## Figures and Tables

**Table 1.** Soil chemical properties in different areas of Yangmingshan National Park*

Soil Chemical Properties	HZS	LSK	ZIH
pH	4.59±0.43^a^	3.76±0.05^b^	4.92±0.12^a^
OM (%)	6.93±2.80^a^	9.61±0.30^a^	6.29±0.70^a^
N (%)	1.14±0.50^a^	0.53±0.01^b^	0.46±0.01^b^
P (ppm)	2.01±0.45^c^	5.10±2.58^b^	13.88±0.14^a^
As (ppm)	16.40±5.94^a^	26.33±0.29^a^	21.87±0.92^a^
Cr (ppm)	0.00±0.00^c^	19.13±0.12^b^	48.33±0.57^a^
Cu (ppm)	127.60±18.99^a^	55.90±1.04^b^	113.93±3.23^a^
Ni (ppm)	26.20±16.38^a^	0.00±0.00^a^	0.00±0.00^a^
Pb (ppm)	36.60±15.10^b^	57.50±0.17^b^	83.13±0.23^a^
Zn (ppm)	108.00±19.63^b^	36.13±0.58^c^	147.73±2.19^a^

* HZS, Huangzuishan; LSK, Lengshuikeng; ZIH, Zhuzihu. Significant difference indicated by different lowercase letters (*P*<0.05).
